# Prediction of Bandgap and Key Feature Analysis of Lead-Free Double Perovskite Oxides Based on Deep Learning

**DOI:** 10.3390/molecules31061032

**Published:** 2026-03-19

**Authors:** Beibei Wang, Juan Wang

**Affiliations:** Xi’an Key Laboratory of Advanced Photo-Electronics Materials and Energy Conversion Device, School of Electronic Information, Xijing University, Xi’an 710123, China

**Keywords:** lead-free double perovskite oxide, bandgap prediction, deep learning algorithm, feature selection, SHAP explanation

## Abstract

Lead-free double perovskites possess the capabilities of wide bandgap control, excellent photoelectric performance, and environmental friendliness. They are an ideal alternative system for addressing the heavy metal toxicity of lead-based perovskites and promoting their large-scale application. Precise control of their bandgap is key to the green transformation of optoelectronic devices. Bandgap, as a key parameter determining the photoelectric properties of materials, has limitations in traditional experimental determination and DFT calculation methods, such as being time consuming, labour intensive, costly, and difficult to achieve high-throughput screening. Deep learning provides an efficient solution to this problem, but current research has issues such as a single-model architecture and poor interpretability, which cannot effectively support bandgap regulation. This study utilised 2367 valid datasets of lead-free double perovskites sourced from the Materials Project database and relevant literature. Following preprocessing steps, including MinMaxScaler normalisation and Pearson correlation coefficient screening, the dataset was divided into a ratio of 7:1:2. The bandgap prediction capabilities of four models—MLP, deep ensemble learning, PINN, and Transformer—were systematically compared, with feature importance analysed using the SHAP method. The results show that the MLP model performs the best in medium-scale, structured feature prediction. The R^2^ value of the test set is 0.9311, while the MAE, MSE, and RMSE are 0.1915 eV, 0.0975 eV^2^, and 0.3122 eV, respectively. A total of 98% of the test samples have a prediction error of ≤0.4 eV, highlighting the stability of low bandgap systems. The Transformer is more suitable for large-scale, sequential feature prediction, while the MLP has limited generalisation ability for medium-to-high bandgap systems containing elements such as Si and Mg. The SHAP analysis revealed that the five electronic structure descriptors, such as B_HOMO+ and A_LUMO+, are the key influencing factors of the bandgap. The research results are helpful for the high-precision prediction and mechanism explanation of the bandgap of lead-free double perovskites, providing theoretical support for rational material design, performance optimisation, and bandgap-oriented regulation. They also point out the direction for subsequent model improvement.

## 1. Introduction

Perovskite materials, due to their high absorption coefficient, excellent carrier transport properties, wide tunable bandgap, and low-cost fabrication advantages, have become the core candidate materials in fields such as photovoltaics, light-emitting diodes, and photoelectric detection, significantly promoting the performance breakthroughs and technological innovations of the next-generation optoelectronic devices [1,2]. Among them, lead-based perovskites (CsPbX_3_, X = Cl, Br, I) have become a research hotspot due to their high luminescence quantum yield and excellent photoelectric conversion performance. However, the heavy metal lead contained in these materials has strong toxicity and poses potential threats to the environment and human health, severely restricting their large-scale commercial application [3]. In this context, the development of lead-free perovskite materials has become a key direction for addressing toxicity issues and achieving sustainable development. Currently, lead-free perovskites primarily consist of two types of systems: single perovskites and double perovskites. Compared with single perovskites, lead-free double perovskites form an ordered lattice structure by introducing two different valence cations. They not only retain the efficient photoelectric properties of traditional perovskites, but also have a wider bandgap control range, better light absorption and carrier transport capabilities, as well as higher chemical stability and environmental friendliness, etc. Therefore, lead-free double perovskites (LFDPs) have become the most promising alternative system to lead-based perovskites at present. The optimisation of their performance and precise control of the bandgap have become the key to promoting the green transformation in fields such as photovoltaics and LEDs, and are also the current research focus in the field of materials science [4].

Bandgap, as a key parameter for evaluating the performance of photovoltaic materials, directly determines the material’s absorption range, carrier separation efficiency, and photoelectric conversion efficiency, and plays a decisive role in the core performance of photovoltaic devices [5]. For materials used in solar cells, an ideal bandgap of 1.2–1.4 eV is required to match the solar spectrum, while the emission wavelength of light-emitting devices is entirely controlled by the bandgap size [6]. Therefore, accurately obtaining and effectively regulating the bandgap of LFDPs is the prerequisite for optimising their application in various photovoltaic fields. However, the traditional methods for determining and calculating the bandgap have two significant limitations. Firstly, the experimental determination requires a complex process involving material synthesis, sample preparation, and performance characterisation, which is not only time consuming and labour intensive but also costly, making it difficult to achieve rapid and large-scale screening of component space [7]. Secondly, traditional computational methods such as density functional theory (DFT) can explain the relationship between material structure and efficacy at the atomic level. However, this method requires a large amount of computational resources and may encounter problems such as high computational cost and long processing time when dealing with large-scale systems and multi-component structures. As a result, it is difficult to quickly complete high-throughput screening of candidate structures, which to some extent limits the development process of LFDPs [8].

In recent years, deep learning technology, with its powerful data mining, nonlinear fitting, and feature adaptive learning capabilities, has been widely applied in the field of bandgap prediction for LFDPs. It has gradually overcome the limitations of the traditional density functional theory (DFT) calculations, which are time consuming and complex, and has become a core tool for accelerating the design and screening of new perovskite materials. Deep learning methods can effectively uncover the potential correlations between material composition, crystal structure, and bandgap. They have significant advantages in prediction accuracy and computational efficiency, providing an efficient path for the performance optimisation of LFDPs and the development of new systems.

Research on machine learning (ML) for predicting the bandgap of perovskite materials has yielded significant results. Numerous classic ML algorithms have been widely applied to this task. For instance, the AdaBoost model achieved a coefficient of determination (R^2^) of 0.9163 and an MAE as low as 0.0283 in predicting the performance of perovskite solar cells [9]. The Support Vector Regression (SVR) model yielded an R^2^ of 0.927 and an RMSE of 0.271 eV for the prediction of the bandgap of ABO_3_ perovskite oxides [10]. The Kernel Ridge Regression (KRR) model obtained an R^2^ value of 0.914 in the training set predicting the bandgap of halides [11]. For HSE-derived bandgaps, the XGBoost model demonstrated an R^2^ score of 0.8008 and an MAE of 0.2848 eV on the test set [12]. Although these traditional models have achieved rapid prediction of the bandgap, there is still room for improvement in terms of prediction accuracy and generalisation ability. Based on this, the data-driven model centred on deep learning provides a completely new paradigm for material performance prediction [13]. Compared with traditional ML methods, the deep learning model can autonomously discover the complex nonlinear relationships between components, crystal structures, and macroscopic properties from massive material data, without relying on explicit physical and chemical mechanism assumptions. Therefore, it has advantages such as high prediction accuracy and high computational efficiency [14,15]. In the current research on material performance prediction, the cross-scale modelling technology based on neural networks has significantly improved the accuracy of bandgap prediction. Saidi [16] employed a hierarchical convolutional neural network (HCNN) to predict the structure and electronic properties of metal halide perovskites (MHPs). Without the need for costly DFT calculations, it achieved highly accurate predictions of lattice constants, octahedral tilt angles, and bandgaps. The root mean square error (RMSE) of the bandgap prediction was as low as 0.02 eV. Stoichev et al. The authors of [17] proposed an artificial neural network optimised by recursive feature filtering, which controlled the prediction error of the three-dimensional perovskite bandgap within 0.25 eV. Qin et al. [18] developed a modelling method that combines graph structure with the Transformer attention mechanism, providing a feasible technical paradigm for the performance prediction of perovskite and other crystalline materials. Although the current research on deep learning for perovskite bandgap prediction has made a series of progress, it has not yet conducted a systematic and in-depth comparative analysis of different deep learning algorithms, and it is impossible to determine the optimal model and applicable scenarios for LFDP bandgap prediction.

Among the numerous deep learning algorithms suitable for bandgap prediction, the multi-layer perceptron (MLP), physical information neural network (PINN), deep ensemble learning, and Transformer, due to their superior performance, have been selected as the core algorithms for this research. Among them, MLP, as a classic deep learning model, possesses extremely strong nonlinear fitting capabilities and can effectively capture the complex mapping relationship between material characteristics and bandgaps. It has demonstrated mature application potential in predicting the bandgaps of perovskite materials. For instance, Mammeri et al. [19] used an MLP to construct a bandgap prediction model, which has excellent generalisation performance. The mean square error in predicting the bandgaps of new materials can be as low as 0.002, which can meet the high-precision prediction requirements under conventional datasets. The core advantage of PINN lies in its ability to embed the energy band theory equations from solid-state physics into the network structure. Without the need for a large amount of labelled data, it can achieve high-precision predictions and is suitable for scenarios with small sample datasets. This advantage can effectively solve the problem of scarce experimental data in some parts of the LFDP systems, such as the improved PINN proposed by Kim et al. [20], which can integrate noisy experimental data and physical constraints, and can complete the prediction of system parameters with only 3 or fewer data points. Its adaptability and practicality are extremely strong. Deep ensemble learning effectively reduces the prediction bias of a single model by integrating the prediction results of multiple base models, thereby enhancing the robustness and stability of the model. Yu et al. [21] constructed the multi-output ensemble deep learning (MOEDL) framework, which combines Bayesian optimisation, attention mechanism, and ridge regression strategies. The prediction results of 10 ionic battery material properties were highly consistent with DFT data. The Pearson correlation coefficient (PCC) and determination coefficient (R^2^) exceeded 0.97 and 0.93, respectively, effectively improving the reliability of LFDP bandgap prediction. The Transformer, due to its powerful global feature-capturing ability through the attention mechanism, can efficiently process high-dimensional material feature data and break through the limitations of traditional algorithms in high-dimensional feature spaces. The general atomic embedding method (ct-UAE), based on the Transformer architecture proposed by Du and Jin [22], not only significantly improves the prediction accuracy of physical properties such as bandgaps, but also its multi-task learning mechanism endows the model with strong generalisation ability in small data tasks, further compensating for the prediction shortcomings in the small sample scenarios. Based on the advantages of various algorithms, it can cover the needs of different scenarios in lead-free double perovskite bandgap prediction, such as regular samples, small samples, and high-dimensional features. Therefore, as the core algorithm of this study, it provides support for the selection of the optimal model and the adaptation of scenarios.

The problem of insufficient and poorly interpretable analysis of key influencing features in bandgap is particularly prominent in the research of bandgap prediction in deep learning, which restricts the theoretical guidance for material component optimisation and bandgap-oriented regulation. Currently, deep learning has become the mainstream technical path due to its excellent nonlinear fitting ability, but such models generally have black box characteristics and insufficient interpretability [23]. Specifically, the existing models can generate prediction results but fail to explain the underlying logic. They are unable to clearly identify the key influencing factors of the bandgap, nor can they quantify the regulatory weights and mechanisms, thus providing no effective theoretical support. To address this bottleneck, introducing the SHAP method to enhance the interpretability of the bandgap prediction model holds significant value. This method is based on the Shapley value principle in game theory and can quantify the contribution of each input feature to the prediction result. It can not only achieve local explanations for individual samples but also conduct global feature analysis, breaking through the limitations of black boxes without sacrificing accuracy. It can effectively make up for the shortcomings of existing methods and provide precise theoretical support for material optimisation and bandgap regulation [24,25].

In view of this, this paper focuses on the bandgap prediction of LFDPs. To address the issues of a single model architecture and insufficient interpretability in existing research, the performance of four deep learning models, namely MLP, deep ensemble learning, PINN, and Transformer, is systematically compared. Combined with the SHAP explainability analysis method, the influence of key material features on the bandgap is quantified, aiming to achieve the dual goals of high-precision prediction and mechanism explanation, providing theoretical support for the rational design and performance optimisation of LFDPs. It also provides a crucial electronic structure feature basis and algorithmic reference for the directional prediction and design of bandgaps under dimensionality control methods, such as quantum confinement effects.

## 2. Results and Discussion

### 2.1. Feature Engineering

This study aimed to predict the bandgap of lead-free double perovskite oxides (LFDP-Os). A feature descriptor system consisting of three dimensions: structure, electronic structure, and performance and stability, was constructed, with a total of 40 feature indicators. The definitions, units, and physical meanings of each descriptor are shown in Table 1, laying the foundation for subsequent feature preprocessing and correlation analysis.

#### 2.1.1. Feature Processing

In this study, exploratory data analysis was first employed to preprocess the dataset, and then the MinMaxScaler method was utilised to perform normalisation and standardization operations on the newly constructed dataset [26]. This method can map the data to a fixed range, effectively eliminating the interference caused by the differences in feature magnitudes, allowing all features to be uniformly scaled to the same magnitude. At the same time, it supports reverse mapping to restore the original data. It cannot only alleviate the problem of scale imbalance among features, promote the fair distribution of different feature weights, but also accelerate the convergence process of the optimisation algorithm, improve the training efficiency of the model, and thereby optimise the performance and stability of the ML model [27]. The normalisation operation process of MinMaxScaler is as follows:

(1) Firstly, determine the minimum value and the maximum value for each feature in the training set;

(2) Subsequently, for each sample’s feature value x, calculate its normalized result using Formula (1);

(1) $$X_{normalize}=(x-min)/(max-min)$$

(3) Finally, the values of each feature are uniformly mapped to the range of [0, 1].

**Figure 1:**
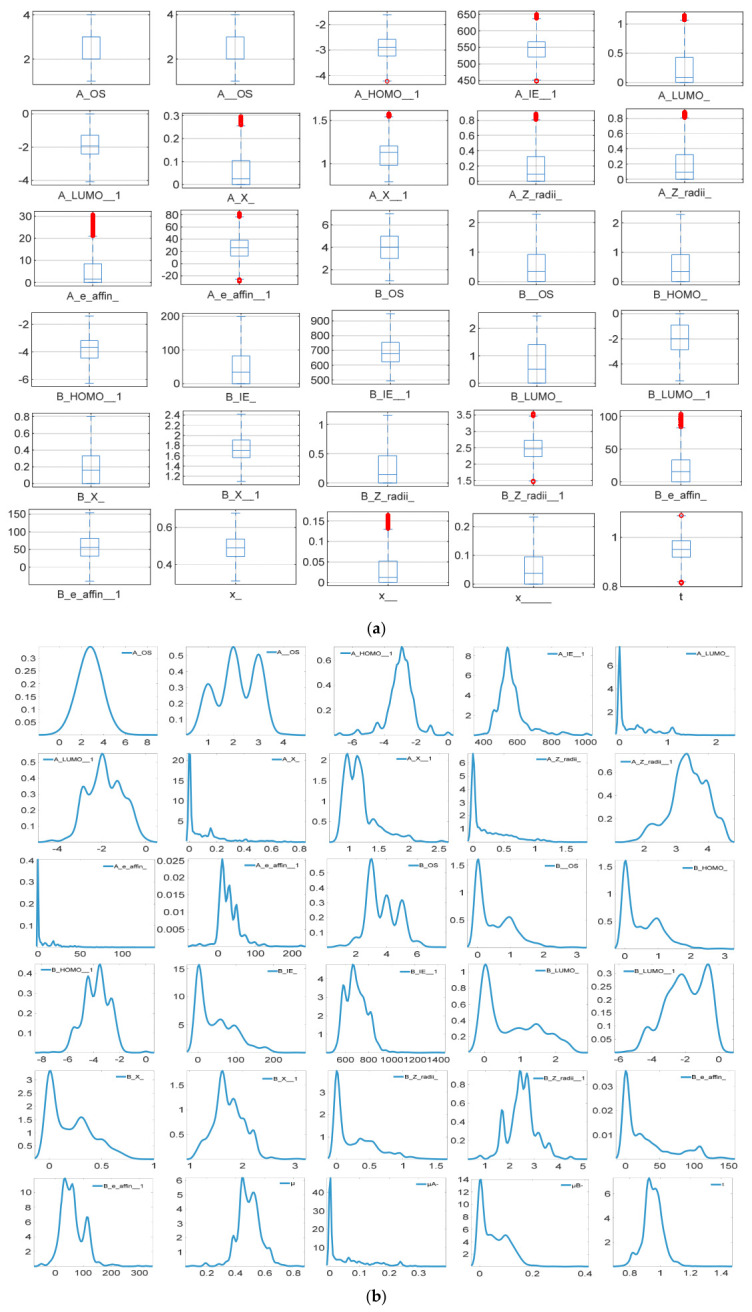
Characteristic box plot and density plot of bandgap of LFDP-Os (**a**,**b**). Red points represent outliers (extreme values) outside the range of the box plot whiskers.

This study employed a combined visualisation analysis method of box plots and density plots to conduct an intuitive analysis of the bandgap characteristics. Among them, the box plot can clearly display the median, quartiles, extreme values, and outliers of the data. The values exceeding the upper and lower limits are marked with circles. By using density plots and box plots to jointly analyse the skewness and other distribution characteristics of the data, in order to achieve a global comparison of multiple features, the coordinate axes are standardised, and all the original features are integrated into the same chart. Figure 1a presents the boxplot distribution of each feature, which visually depicts the numerical discrete characteristics and outlier situations of different attributes. As can be seen from the figure, the numerical ranges of each feature vary significantly. Some features (such as A_LUMO−, B_X−) have their values concentrated within a narrow range from 0 to 1, while features like A_E+ and B_E+ have numerical ranges extending to hundreds of orders of magnitude. Features such as A_HOMO+ and B_HOMO+ also include negative values. This difference is in line with the inherent magnitude differences of the physical or chemical properties corresponding to each feature. Meanwhile, there are obvious outliers (marked in red) in features such as A_E+, A_e_affin−, B_z_radii+, and B_e_affin−. The corresponding attribute values of these samples deviate significantly from the overall quartile range, indicating that they might be samples with unique structures or properties. In the subsequent analysis, it is necessary to distinguish whether these outliers are noise interference or truly extreme attribute samples. The density plots of the distribution of each feature are shown in Figure 1b. Through the analysis of the density curves, the distribution patterns and skewness characteristics of each feature in the dataset can be determined. The density curves of most features show a multi-peak distribution, indicating the presence of different data distributions. The density curves of the descriptors A_LUMO−, A_X−, A_Z_radii−, A_e_affin−, and mismatch factor in the A position element show a clearly left-skewed shape, indicating that the data distribution is skewed. The density curve of the A_OS descriptor is a single-peak symmetric distribution, suggesting that the original data distribution corresponding to the feature is relatively uniform.

#### 2.1.2. Feature Association Analysis

Feature selection techniques play a crucial role in the field of ML. The core objective is to identify the most relevant or representative subset of features from the raw data, which can effectively reduce the data dimension, eliminate irrelevant or redundant features, thereby improving the accuracy and generalisation ability of the model, and enhancing the interpretability of the model [28]. During this process, the Pearson correlation coefficient is often used to assess the correlation between each feature and the target variable, and features with a strong correlation with the target variable are selected. The Pearson correlation coefficient is a statistical measure that quantifies the strength of the linear relationship between two variables, and is commonly used to evaluate the degree of correlation between features. Its definition is as shown in Formula (2).

(2) $$\rho_{X,Y}=\frac{cov(X,Y)}{\sigma_{X}\sigma_{Y}}=\frac{\sum_{i=1}^{n}\left(X_{i}-X^{-})(Y_{i}-Y^{-}\right)}{\sqrt{\sum_{i=1}^{n}{\left(X_{i}-X^{-}\right)}^{2}\sum_{i=1}^{n}{\left(Y_{i}-Y^{-}\right)}^{2}}}$$

**Figure 2:**
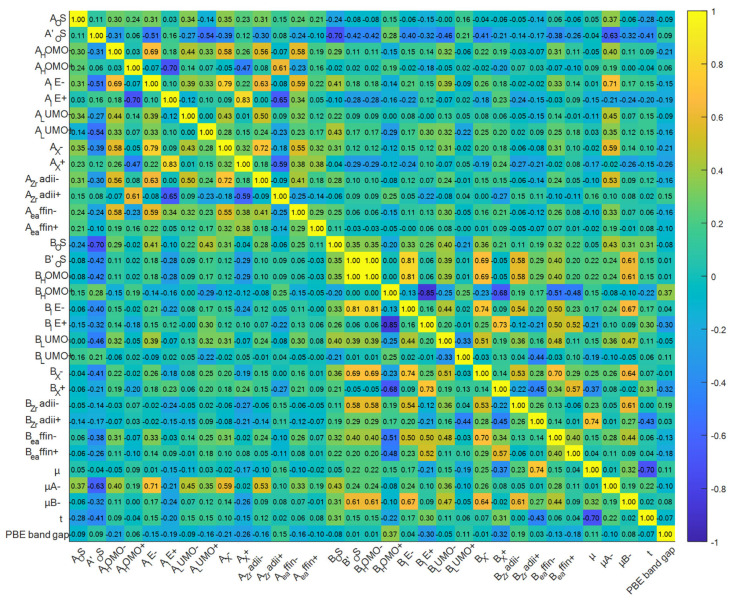
Pearson correlation analysis of bandgap characteristics.

This paper uses a Pearson correlation coefficient with a range from −1 to 1 to evaluate the correlation between each feature and the target variable; −1 indicates a completely negative correlation, 1 indicates a completely positive correlation, and 0 indicates no linear correlation. Strongly correlated features are selected to eliminate redundant variables, reduce data dimensions, and improve model performance. The dataset is initially screened based on the derived correlation coefficients, and the feature Pearson correlation graph is shown in Figure 2. The gradient colour bar on the right side of the figure is divided according to the magnitude of the correlation coefficient. Yellow indicates a positive correlation, and blue indicates a negative correlation. The darkness of the colour is positively correlated with the strength of the correlation. The results show that the correlation coefficient between B_HOMO− and B’_OS is 1.0000, and the correlation coefficient between B_IE+ and B_HOMO+ is −0.8536, indicating a strong negative correlation between the two. This study sets the absolute value of the correlation coefficient ≥ 0.8 as the threshold for strong correlation, and identifies multiple groups of strong correlation features, such as A_IE+ and A_X+, B_HOMO+ and B_IE+.

**Figure 3:**
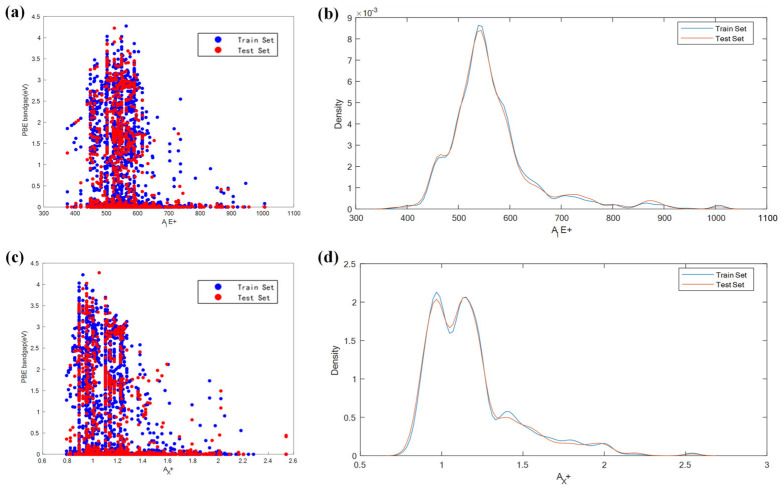
(**a**,**c**) respectively represent the correlation distribution of the A_IE+ and A_X+ features and the bandgap in the training set and test set; (**b**,**d**) correspond to the value density distribution of the two features.

As shown in Table 2, when the Pearson correlation coefficient between two features approaches 1, it indicates that when one feature increases, the other feature also increases almost proportionally. This usually leads to the problem of multicollinearity. The existence of multicollinearity can adversely affect the performance of linear models that rely on estimated coefficients. In such cases, the stability of the model may be compromised, resulting in unreliable coefficient estimates and reduced interpretability [29]. The correlation coefficients between A_IE+ and A_X+, between B_HOMO− and B_IE−, and between B_HOMO+ and B_IE+ reached 0.8330, 0.8050, and 1.0000, respectively, indicating that the two features in these three groups have a very high linear correlation. To reduce the dimensionality of the data, removing these highly correlated elements is considered. The method for deleting highly correlated features is as follows: when the Pearson correlation coefficient between feature A and feature B is close to 1, select one of the features and construct a feature set with the target value for prediction. Then, compare the R^2^ values of the prediction models in the cases of feature A and feature B, and choose the case with the higher R^2^ value. At the same time, MSE and MAE should also be taken into consideration. After deleting a highly correlated feature, MSE is used to determine whether the model is overfitting or underfitting. Figure 3 shows the correlation patterns and sample distribution characteristics of feature A_IE+, A_X+ and bandgap in the training set and test set. In feature selection, by comparing the performance of the two features, the scatter plot distribution corresponding to A_X+ is more concentrated and the correlation trend is clearer (Figure 3c), while the bandgap fluctuation of A_IE+ is greater (Figure 3a); from the sample distribution perspective, the density curves of the training set and test set of A_X+ are highly overlapping (Figure 3d), and the consistency of the distribution is better than that of A_IE+ (Figure 3b). Based on the stability of the correlation between the above features and the target, as well as the rationality of the sample distribution, feature A_X+ is retained. Additionally, the key characteristics that affect the bandgap need to be considered. Although B’_OS, B_IE−, and B_HOMO− are highly correlated, the ionisation energy of the B position element B_IE− and the highest occupied molecular orbital energy of the B position element are strongly correlated with the bandgap of the compound. Therefore, the feature B’_OS is excluded. B_HOMO+, B_HOMO−, and B_IE+ are core parameters of the double perovskite electronic structure and are directly related to the bandgap. Thus, they are retained. The final selected feature set not only retains the core information but also eliminates redundant interference, providing a more concise and effective variable basis for subsequent analysis.

### 2.2. Model Prediction Results

Four deep learning algorithms were used for training and testing the dataset—MLP, deep ensemble learning algorithm, the physical information neural network (PINN), and the Transformer architecture. The range of the coefficient of determination R^2^ is [0, 1]. The closer the value is to 1, the better the predictive performance of the model. The mean absolute error (MAE) is more sensitive to small prediction deviations, while the mean square error (MSE) is more sensitive to large prediction deviations. The performance of the prediction model is shown in Table 3. On the R^2^ dimension, the MLP (0.9311) performed the best, while the Transformer (0.8178) was relatively weaker. In terms of error metrics, the MAE (0.1915), MSE (0.0975), and RMSE (0.3122) of the MLP were the smallest among all four, and the error metrics of the deep ensemble learning model were also at a relatively low level. However, the error data of PINN and Transformer were significantly higher. To further verify the superiority of the deep learning model in predicting the bandgap of LFDPs, this paper quantitatively compares the optimal deep learning model (MLP) with the traditional ML models (AdaBoost, SVR, KRR, XGBoost) applied in this field from the literature 9–12. From the core indicators, the R^2^ of the MLP model in this paper is 1.61%, 0.44%, 1.87%, and 16.27% higher than those of AdaBoost, SVR, KRR, and XGBoost, respectively. Overall, the deep learning MLP model demonstrates stronger fitting ability in regression tasks and meets the industrialisation requirements of high-throughput screening.

From the perspective of model adaptability, in the prediction of the bandgap of LFDP, MLP performs the best while Transformer performs poorly. The core issue is that there is a significant difference in the adaptability of the two to the task scenario: Firstly, the parameter and data volume adaptability are different. The Transformer model has an approximate 120,000 parameter scale, which is not well suited for the small sample data in this study. The self-attention mechanism cannot function properly and is prone to overfitting, while the parameter efficiency of the MLP model is in line with the existing data scale, which can effectively avoid overfitting. Secondly, there is a difference in the compatibility between the tasks and the architecture. Bandgap prediction relies on low-dimensional structured features, while the attention mechanism of the Transformer will introduce redundant computations, thereby increasing the prediction error. The fully connected MLP architecture can efficiently capture the nonlinear relationship between features and bandgap, and its mapping advantage is more prominent. Thirdly, the adaptability of regularisation and small sample training is different. MLP adopts a combined regularisation strategy, which is more suitable for small sample training scenarios than the single regularisation of Transformer, and can improve the stability of the validation set loss. This performance differentiation is consistent with the research conclusions in related fields, verifying the optimality of MLP in low-dimensional small sample material modelling, and providing a reliable reference for model selection in similar low-dimensional small sample material modelling tasks.

**Figure 4:**
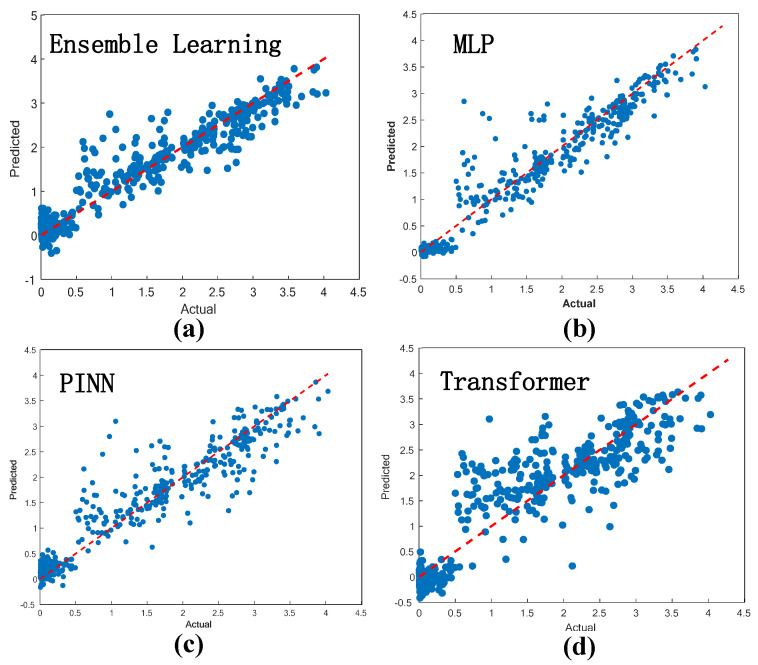
Regression model for bandgap prediction of LFDP-Os based on deep learning algorithms. (**a**) Fitting a graph based on a deep ensemble learning model. (**b**) Fitting the graph based on the MLP model. (**c**) Fitting the graph based on the PINN model. (**d**) Fitting a graph based on the Transformer model. The red dashed line in each subplot represents the ideal prediction line (Predicted = Actual), where predicted bandgap values perfectly match the actual values.

In Figure 4, the horizontal axis represents the actual value of the bandgap, which is obtained through DFT calculation; the vertical axis indicates the bandgap value predicted using deep learning. In this study, four deep learning algorithms were selected to predict the bandgap values of LFDP-Os, and the predicted results were compared with the calculated values from DFT. The results show that the predicted values of the four algorithms exhibit a nearly linear relationship with the DFT calculated values, which indicates that the deep integration, MLP, PINN, and Transformer all can achieve good fitting and prediction of the bandgap values of LFDP-Os.

From the prediction results of the MLP model on the first 20 groups of samples in the test set shown in Table 4, it can be seen that the absolute values of the overall bandgap prediction errors of the model are distributed within the range of 0.0129 to 0.9020 eV. Among them, the absolute values of the errors of 16 groups of samples do not exceed 0.4 eV, accounting for 80%, indicating that the model has good fitting accuracy for the bandgap prediction of most double perovskite systems. In the application scenarios of solar cells, the device performance is usually optimal when the bandgap is within the range of 1.2–1.4 eV [30]. To evaluate the predictive ability of the model in this critical range, we selected the samples from the test set that are closest to this range (1.5–1.8 eV) for analysis. The results show that the predicted errors for Ba_2_ErBiO_6_ and Ba_2_BiDyO_6_ are only 0.0201 eV and 0.0453 eV, respectively, both of which are significantly smaller than 0.1 eV. The error for AgTaO_3_ is controlled within 0.2 eV, indicating that the model has high reliability within the practical bandgap range.

Meanwhile, there are still a few samples in the test set with relatively large prediction deviations. The predicted bandgap of AsBO_3_ is approximately 1.80 times that of the PBE bandgap, while the predicted value of Ba_2_InSbO_6_ is only 19% of the corresponding PBE value. Both show significant deviations. By further analysing the structure of the dataset and the distribution of elements, these significant deviations mainly result from the insufficient number of samples of the corresponding components in the training set and the lack of diversity in the coordination environment of the elements, making it difficult for the model to fully learn the correlation between the electronic structure and the bandgap of these components, leading to a decline in local prediction accuracy. Similarly, Ba_2_MgTeO_6_ also exhibits a significant error due to the insufficient representation of the Mg element in the training set, which further confirms this reason.

Overall, the MLP model still maintains high prediction accuracy and practical value in the key bandgap range of solar cells. Meanwhile, the prediction deviations of a few systems have pointed out the direction for the subsequent expansion of the dataset, optimisation of feature engineering, and improvement of the model structure.

To further clarify the prediction accuracy and stability of the MLP model in different bandgap intervals, the test set was divided into four groups based on the bandgap range: low bandgap, medium–low bandgap, medium–high bandgap, and high bandgap. The error indicators of each group are shown in Table 5. From the results, it can be seen that the MLP model exhibits excellent predictive performance across all bandgap intervals. The error indicators in each interval show a slight fluctuation pattern: for the low bandgap group (214 samples), the MAE is 0.1915 eV, the MSE is 0.0975 eV^2^, and the RMSE is 0.3122 eV; 91.12% of the samples have a prediction error of ≤0.4 eV. For the medium–low bandgap group (97 samples), the MAE is 0.1922 eV, the MSE is 0.0979 eV^2^, and the RMSE is 0.3128 eV; the proportion of samples with an error ≤0.4 eV is 81.44%. For the medium–high bandgap group (153 samples), the MAE is 0.1932 eV, the MSE is 0.0990 eV^2^, and the RMSE is 0.3146 eV; 86.27% of the samples meet the requirement of ≤0.4 eV; for the high bandgap group (9 samples), due to the small sample size, the prediction consistency slightly decreases, with an MAE of 0.1942 eV, an MSE of 0.0983 eV^2^, and an RMSE of 0.3135 eV; the proportion of samples with an error ≤0.4 eV is 66.67%. Overall, the MLP model shows stable prediction errors in each bandgap range. The prediction accuracy is the highest in the low bandgap range (91.12%), followed by the medium–high and medium–low bandgap ranges. The high bandgap range performs relatively weakly due to the small sample size. The model’s overall prediction for the bandgap of LFDP-Os is reliable, with only a few deviations in the medium–low and high bandgap ranges. This is related to the insufficient characterisation of some special element systems and the small sample size. Further optimisation of the prediction performance can be achieved by introducing fine structural descriptors or transfer learning.

### 2.3. Feature Importance Analysis

To address the black-box issue of ML models and enhance the credibility and interpretability of the bandgap prediction results, this paper introduces the SHAP (SHapley Additive exPlanations) analysis method based on the Shapley value principle in game theory [31]. This method overcomes the shortcomings of other interpretation methods. It can not only decompose the local single-sample prediction results, but also complete the feature contribution analysis at the global model level. Its core is to decompose the sample prediction output into the average value of all sample predictions and the sum of each feature’s SHAP value. The positive or negative nature of the SHAP value is used to determine the positive or negative effect of the feature on the gap, and its absolute value is used to measure the influence intensity [32]. During implementation, it is necessary to first build an adapter for the SHAP interpreter, input the test set to calculate the SHAP values for each sample feature, and then through visual results such as summary graphs and dependency graphs, quantify the global and local impacts of each feature, identify the key features that have the greatest impact on the bandgap prediction task, and simultaneously clarify the interaction and value influence patterns between features, effectively clarify the prediction logic of tree-based regression models, and assist in handling missing values in tree models to improve the accuracy and robustness of the interpretation, which not only helps to understand how the model uses input features to generate bandgap prediction results, but also enables the combination of perovskite electronic structure and band theory to verify the physical essence of the influence of features, providing a theoretical basis for improving model performance and experimental-level bandgap control.

**Figure 5:**
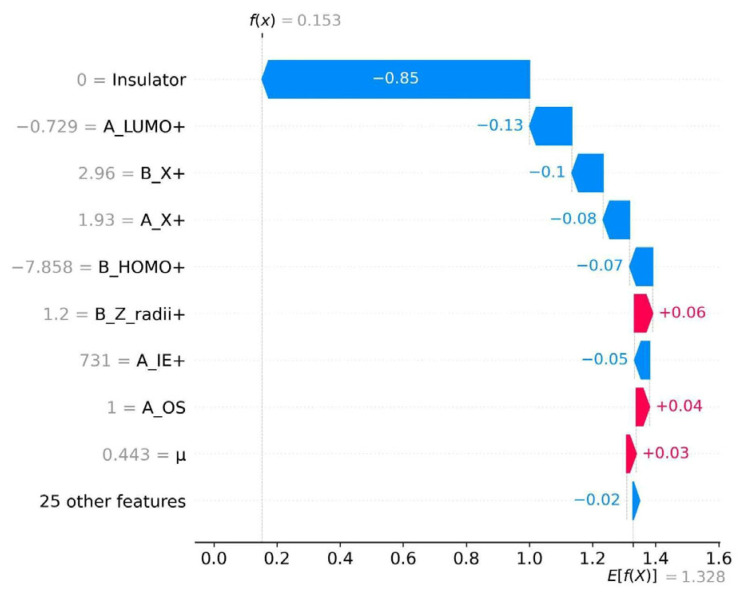
Correlation analysis of bandgap prediction and input features. In the figure, the blue color represents the negative contribution of the features to the prediction result, while the red color represents the positive contribution.

Figure 5 presents the SHAP feature importance graph in descending order of contribution, highlighting ten key features that intuitively show the contribution of each feature to the model’s prediction results. Negative values correspond to negative contributions, and positive values correspond to positive contributions. It should be noted that the feature values are inversely correlated with the positivity/negativity of the prediction results—although the red markers indicate the positive SHAP influence of the features, when the values of such features increase, they instead have a negative effect on the prediction results, thereby making it more likely to obtain negative prediction results. The SHAP values on the *x*-axis correspond to the names of each feature listed on the *y*-axis. As shown in Figure 5, the prediction of the bandgap of the LFDP-Os is mainly dominated by the five core electronic structure characteristics of B_HOMO+, A_LUMO+, B_X+, A_X+, and A_IE+. The combined contribution of these five descriptors is much higher than that of the other 25 features. They are highly consistent with the band formation mechanism and crystal orbital hybridisation rules of LFDPs, and are also the core physical reasons for being the key influencing factors of the bandgap.

The crystal structure of the LFDPs is of the AA’BB’O_6_ type. Based on the band theory and existing research conclusions, the physical essence of the bandgap is the energy difference between the valence band top (VBM) and the conduction band bottom (CBM) [33]. The energy levels of VBM and CBM are mainly determined by the hybridisation of the A/B site cations with the O2- orbitals. The five core characteristics regulate the energy levels of VBM and CBM from the perspectives of orbital energy levels, hybridisation, chemical bond polarity, and electron binding energy, thereby determining the bandgap size. B_HOMO+ determines the VBM energy level by regulating the hybridisation of the O-2p orbitals and the d orbitals of the B-site cation, directly controlling the lower limit of the bandgap, and the higher its value, the smaller the bandgap [34]. A_LUMO+ mediates the hybridisation of the A-site ion orbitals and the O-2p orbitals to regulate the CBM energy level, and the higher its value, the larger the bandgap [35]. A_X+ and B_X+ can change the polarity of the A-O and B-O bonds, and the enhancement of polarity will cause the VBM to shift downward and the CBM to shift upward, thereby widening the bandgap in both directions [36]. A_IE+ indirectly affects the stability of the CBM by regulating the electronic binding energy and delocalisation characteristics of the ions at position A. The above five features do not act independently but achieve precise control of the bandgap through a coupling mechanism [37]. The combined contribution of the remaining 25 features is relatively weak, further verifying the correlation between the core features and the bandgap, indicating that by prioritising the regulation of the HOMO energy level of the B-site cations, combined with specific electronegativity and ionic radius of the A-site cations, the bandgap can be precisely optimised. This also confirms the rationality and reliability of the feature selection in the bandgap prediction model.

**Figure 6:**
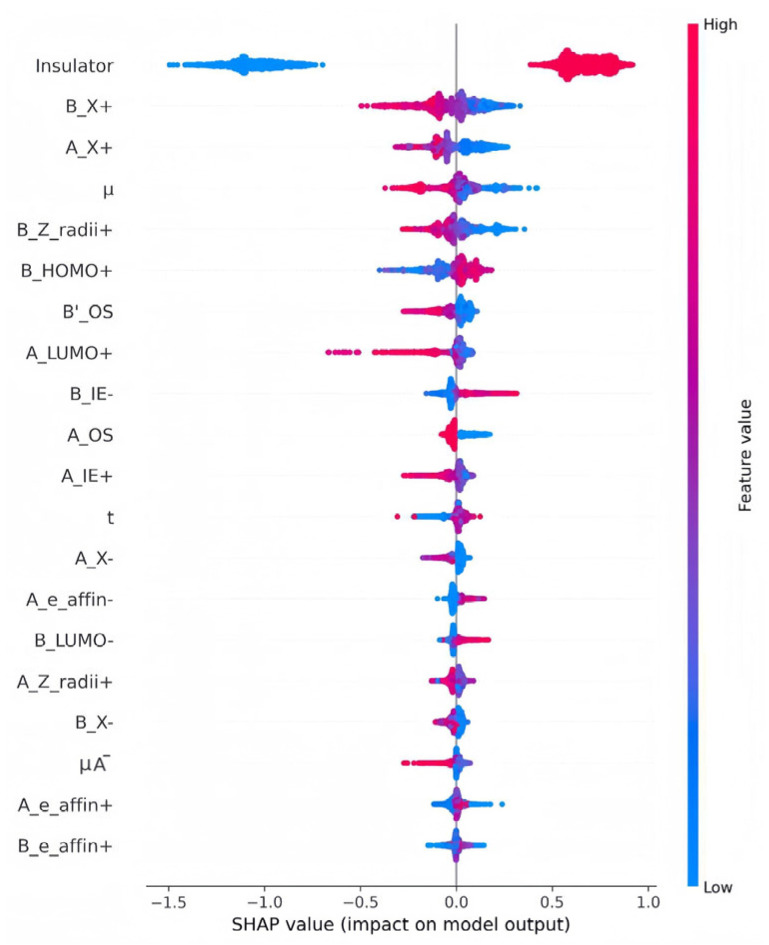
SHAP influence distribution map of the bandgap prediction model for LFDP-Os.

The SHAP feature importance analysis results in Figure 6 indicate that there is a significant nonlinear correlation between each material feature and the bandgap of the perovskite. Moreover, the influence degree and direction of different features on the bandgap prediction are significantly different. Among them, the features such as B_X+, A_X+, and μ exhibit positive driving factors for bandgap prediction at high values, while the features such as B_e_affin+ and A_e_affin+ are key negative regulatory factors at high values; and the SHAP values of features such as B_Z_radii+ and B_HOMO+ have both positive and negative ranges, reflecting the heterogeneity of the impact on the bandgap, and their effect directions will change with the variation of feature values. From a physical perspective, the essence of this kind of nonlinear correlation lies in the fact that when the characteristic values exceed the critical range, the crystal structure and orbital hybridisation mechanism of the perovskite change. This discovery not only quantifies the contribution of each characteristic to the model output but also combines the band theory to reveal the physical essence of the non-monotonic and nonlinear mapping relationship between the characteristics and the bandgap, clarifying the intrinsic logic of the model decision making. It provides a clear priority direction for the optimisation of the subsequent perovskite material bandgap prediction model and the targeted design of experimental-level regulation.

## 3. Materials and Methods

### 3.1. Dataset Preparation

#### 3.1.1. Data Sources

**Figure 7:**
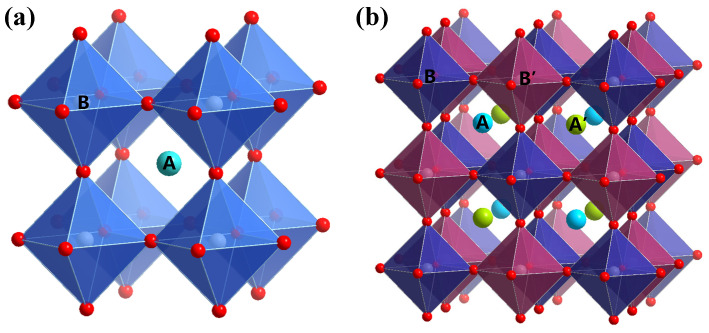
The crystal structure of a single ABO_3_-type perovskite is shown in (**a**), and the crystal structure of a double perovskite AA’BB’O_6_ is shown in (**b**). In this structure, each of the A-sublattices and B-sublattices contains two types of cations [40].

The dataset of lead-free double perovskite oxides (LFDP-Os) used in this study was sourced from the open database of the Materials Project and related literature [38]. After data screening, duplication removal, and feature supplementation, a total of 2367 valid data points were retained. From the perspective of material structure, the double perovskite oxides included in the dataset exhibit a typical multi-octahedral arrangement, as shown in Figure 7. The A-site ions consist of metal cations such as Ba and Pr, while the B-site ions include transition metal cations such as W and Ta. The use of non-lead elements ensures the environmental friendliness of the materials and covers a wide range of structural types. In terms of the feature dimensions of the dataset, there are a total of 40 descriptors. The LFDP-Os bandgap feature descriptor is shown in Table 1 of Section 2.1, covering three major dimensions: structure, electronic structure, and performance and stability, which support multi-dimensional performance analysis. To ensure the scientificity of model training and validation, this study divided the dataset into a training set, validation set, and test set, which were used for parameter optimisation, feature learning, and prediction accuracy evaluation. During the division, the features and bandgap values were kept consistent to avoid the influence of data bias on the model performance [39].

#### 3.1.2. Dataset Division

During the model training and validation stages, in order to ensure the scientificity and reliability of the experimental results, considering the actual situation of the 2367 medium-sized sample size in this study, and referring to the mainstream research practices in the field of ML and the conventional principles of dataset division, this study adopts the stratified sampling method to divide the dataset into training set, validation set, and test set in a ratio of 7:1:2. The training set contains 1657 samples, the validation set contains 237 samples, and the test set contains 473 samples. This division strictly maintains the distribution consistency of each subset with the original dataset in terms of features and gap values, effectively avoiding the adverse impact of data bias on the model performance evaluation. At the same time, it ensures that the training set has an adequate sample size to support model parameter optimisation and avoid the problem of underfitting. It also makes the validation set suitable for hyperparameter tuning and intermediate performance verification, and facilitating timely correction of training deviations. Moreover, through a reasonable proportion of the test set, it can objectively and accurately evaluate the model’s generalisation ability and prediction accuracy, further ensuring the rigour of the model training and validation process [41]. For the double perovskite bandgap prediction task, the four models involved—deep ensemble learning, MLP, PINN, and Transformer—all follow this division strategy. The core principle is to ensure that the validation set and the test set are completely independent of the training process and that the division ratio is 1. The training set is used for model parameter learning, the validation set is used for hyperparameter tuning and the implementation of early stopping strategies, and the test set is used for final performance evaluation [42]. In terms of specific operations, the deep integrated learning, MLP and PINN models randomly assign sample indices through the dividerand function. The Transformer model generates random index sequences manually and slices to complete the division. PINN and MLP additionally add a step of filtering numerical feature columns to adapt to non-numerical features. All models maintain a one-to-one correspondence between the feature matrix and the label vector index to prevent sample and label misalignment. During the training phase, the Transformer and MLP randomly shuffle the training set in batches, while the validation set and test set remain fixed to ensure the comparability of the validation results for each epoch.

### 3.2. Prediction Model

#### 3.2.1. MLP Model

**Figure 8:**
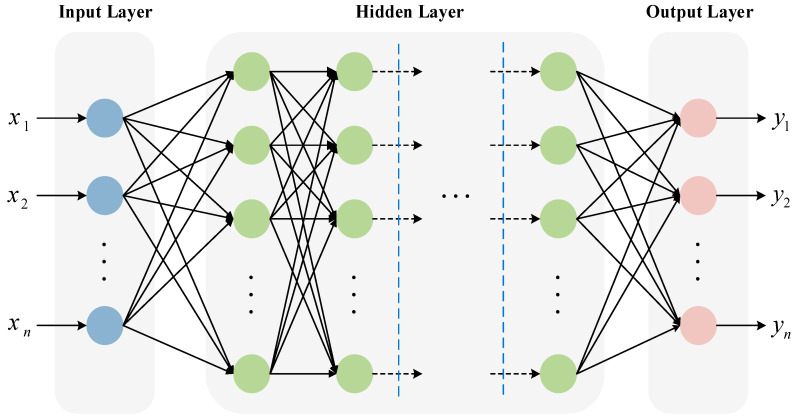
Multi-layer perceptron (MLP) neural network architecture. The ellipsis is used to indicate that the number of hidden layers and the number of neurons in each layer can be flexibly adjusted, demonstrating the scalability of the fully connected network.

The multi-layer perceptron (MLP) is a classic feedforward fully connected neural network in the field of deep learning. It consists of an input layer, at least one hidden layer, and an output layer, and the neurons between layers are connected in a fully connected manner [43]. As shown in Figure 8, its core operation mechanism is divided into two stages: forward propagation and backward propagation. During forward propagation, the input data are weighted summed by each layer of neurons, combined with bias, and transformed by a nonlinear activation function before being passed layer by layer to the output layer to generate the prediction result. Backward propagation, based on the gradient descent algorithm, iteratively updates the weights and bias parameters by backpropagating the error of the loss function to minimise the prediction error. Among them, the activation function is the key component for achieving nonlinear fitting. As a deep learning model, MLP possesses the universal approximation property. In theory, it can achieve high-precision fitting of any nonlinear function by adjusting the depth or width of the hidden layers. It not only has a simple structure and is easy to implement, but can also be directly applied to ML tasks such as classification and regression. Moreover, it is the core basic module of advanced deep learning architectures like convolutional neural networks (CNN) and recurrent neural networks (RNN).

#### 3.2.2. Deep Ensemble Learning Model

**Figure 9:**
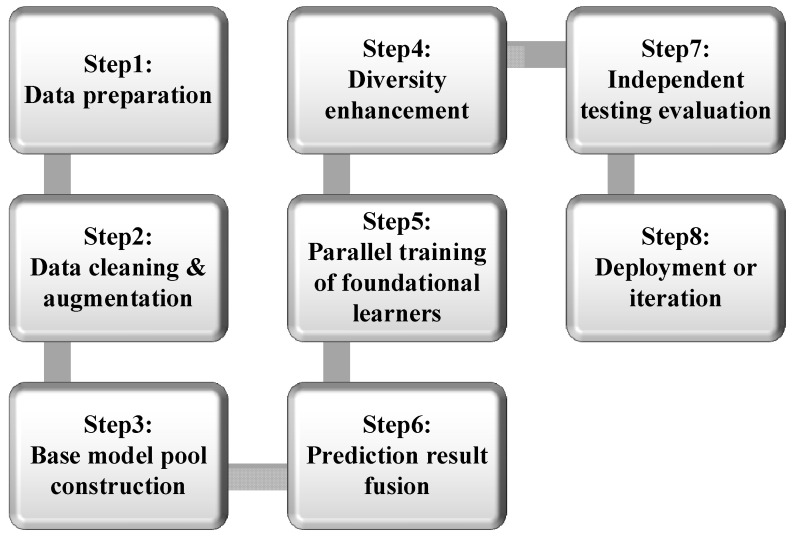
Steps of deep ensemble learning algorithm.

The deep ensemble learning model combines the strong feature extraction capability of deep learning with the high generalisation stability of ensemble learning. Its core lies in constructing multiple structurally differentiated deep learning models as base learners [44]. The algorithm flow is shown in Figure 9. It separately explores the deep nonlinear correlations between the characteristics of perovskite element composition, crystal structure, electronic structure, etc., and the bandgap value. By integrating the prediction results of each sub-model through a specific fusion strategy, the generalisation performance and robustness of the model are enhanced, and the risk of overfitting is reduced. The key components include base learners, training strategies, and fusion strategies. In terms of fusion strategies, for regression tasks, simple averaging (Formula (3)) or weighted averaging (Formula (4)) are commonly used. For classification tasks, the prediction votes for each category can be counted through statistical base learners to determine the final category, which can be divided into hard voting and soft voting. For more complex scenarios, stacking methods can be employed, using meta learners to learn the mapping relationship between the prediction results of base learners and the true labels (Formula (5)), or boosting methods by iteratively updating sample weights (Formula (6)) to optimise the model. Finally, the model performance is evaluated based on indicators such as RMSE, MAE, and R^2^, and further improved prediction accuracy and robustness are achieved through methods such as regularisation, data augmentation, and optimising the diversity of base learners.

(3) $$F(x)=\frac{1}{M}\sum_{i=1}^{M}f_{i}(x)$$

(4) $$F(x)=\sum_{i=1}^{M}w_{i}f_{i}(x)\ldots \sum_{i=1}^{M}w_{i}=1\;\mathrm{and}\;w_{i}\geq 0$$

(5) $$F(x)=g(f_{1}(x),f_{2}(x),\ldots ,f_{M}(x))$$

(6) $$w_{i}^{t+1}=w_{i}^{t}\cdot \exp(\alpha_{t}\cdot I(y_{i}\neq f_{t}(x_{i})))$$

#### 3.2.3. PINN Algorithm

**Figure 10:**
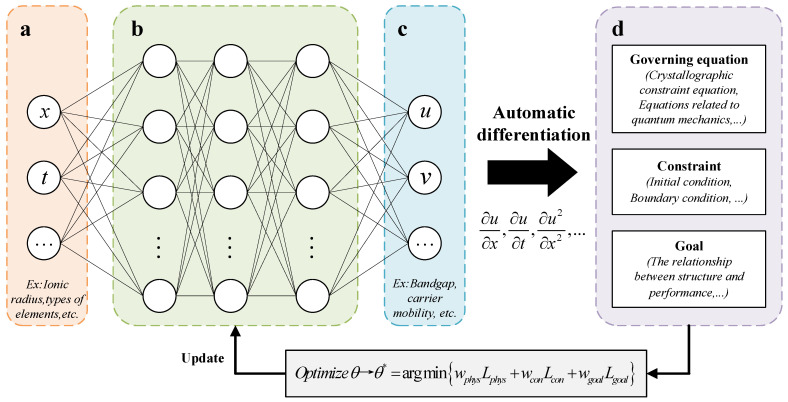
Schematic diagram of the PINN architecture. (**a**) Input of the neural network. (**b**) The target function (θ) to be optimised is composed of multiple layers of perceptrons. (**c**) Output of the neural network. (**d**) The loss function is usually composed of multiple terms, each weighted by different coefficients. These loss functions are weighted and summed to form the final target function. The ellipsis in the figure represents the scalability of the network structure, and θ* represents the optimal parameters after model optimization.

The core principle of PINN (Physical Information Neural Network) is to combine the parameters of perovskite crystal structure, microscopic physical laws, and limited data to construct a predictive model that possesses both data fitting capabilities and physical interpretability [45]. As shown in Figure 10, in practical applications, usually the structural parameters, such as ionic radius, chemical bond parameters, and composition ratio, are taken as inputs, and the key performance indicators, such as bandgap and carrier mobility, are taken as outputs. Based on the universal approximation property of neural networks, a mapping relationship between structure and performance is established. At the same time, the derivatives of the output with respect to the input are solved using automatic differentiation technology. The inherent physical rules of perovskite, such as the quantum mechanics energy band theory, the crystal chemical tolerance factor criterion, and the thermodynamic formation energy constraint, are transformed into physical loss terms; then, combined with the data loss terms of the fitted measured data, a weighted composite loss function is constructed [46]. After end-to-end training, the model can not only fit the known data but also strictly follow the physical essence of perovskite materials. Eventually, it can accurately predict the target performance of unsynthesised perovskite materials, providing support for high-throughput material screening and targeted design.

#### 3.2.4. Transformer Model

The core of the Transformer model lies in the self-attention mechanism, which can precisely capture the dependency relationships between elements at any position within the input sequence by calculating the attention weights of each element in the sequence. This mechanism breaks the limitations of traditional recurrent neural networks in sequence modelling [47,48]. The advantage of this model lies in its ability to handle ordered sequences and global dependencies of large-scale data. However, the characteristics of this study are unstructured and semi-structured attributes, and the sample data are limited, which may restrict its performance. Further adaptation to the task is required by optimising hyperparameters or adding structural descriptors. In the task of predicting the bandgap of perovskite materials, the crystal structure is usually transformed into an ordered sequence using sine and cosine positional encoding (Formula (7)). The model conducts weighted learning on key information such as bonding interactions between atoms and spatial distances through the self-attention mechanism (Formula (8)). Without the need for manual design of complex structural features, it can autonomously discover the potential correlations between atomic arrangement and bandgap values. Among them, pos represents the position index of the atoms in the sequence; i is the dimension index of the position encoding vector; dmodel is the feature dimension of the model. *Q*, *K*, and *V* are obtained by multiplying the input feature vectors by the learnable weight matrices *W_Q_*, *W_K_*, and *W_V_* respectively. *d_k_* is the dimension of *K* and *Q*.

(7) $$\begin{array}{l} PE_{\left(pos,2i\right)}=\sin(\frac{pos}{{10,000}^{2i/d_{\mathrm{mod}el}}})\\ PE_{\left(pos,2i+1\right)}=\cos(\frac{pos}{{10,000}^{2i/d_{\mathrm{mod}el}}}) \end{array}$$

(8) $$Attention(Q,K,V)=soft\max(\frac{QK^{T}}{\sqrt{d_{k}}})V$$

The Transformer adopts a dual-encoder–decoder structure framework. The encoder is composed of multiple layers of self-attention layers and feedforward neural networks, and the layers are connected through residual connections and layer normalisation to ensure training stability [49]. After the input sequence is transformed into vectors through the embedding layer, it is first captured in parallel from different dimensions by the multi-head self-attention layer to capture the interaction features between atoms. Then, it undergoes a nonlinear transformation and integration by the feedforward network (Formula (9)). Finally, the output global feature vector can be directly connected to the fully connected layer to achieve end-to-end prediction of the bandgap value of perovskite. Among them, max(0,x) represents the ReLU activation function, while *W*_1_, *b*_1_, *W*_2_, and *b*_2_ are learnable parameters.

(9) $$FFN(x)=\max(0,xW_{1}+b_{1})W_{2}+b_{2}$$

### 3.3. Valuation Criteria

The performance of the regression model can be evaluated using various metrics [50]. The most common ones include mean squared error (MSE), root mean squared error (RMSE), mean absolute error (MAE), and coefficient of determination (R^2^), which correspond to Formulas (10)–(13). The mean squared error measures the sum of the squares of the differences between the predicted values and the true values. The root mean squared error is its square root. The advantage is that it is proportional to the size of the regression target and is easier to understand. The mean absolute error measures the average of the absolute values of the differences between the predicted values and the true values. These two types of indicators can both measure the prediction error. The difference is that the mean squared error is more sensitive to large prediction errors, while the mean absolute error is more sensitive to small prediction errors. R^2^ is used to measure the degree to which the model explains the changes in the data, and it represents the degree of linear correlation between the predicted values and the actual values. Its range of values is from 0 to 1, with a higher value indicating a stronger explanatory ability of the model.

(10) $$MSE=\frac{1}{N}{\sum_{i=1}^{N}\left(y_{i}-y\right)}^{2}$$

(11) $$MAE=\frac{1}{N}\sum_{i=1}^{N}|y_{i}-y|$$

(12) $$RMSE=\sqrt{\frac{1}{N}{\sum_{i=1}^{N}\left(y_{i}-y\right)}^{2}}$$

(13) $$R^{2}=1-\frac{\sum_{i=1}^{N}(y_{i}-y{)}^{2}}{\sum_{i=1}^{N}(y_{i}-{\bar{y}}_{i}{)}^{2}}$$

Among them, *y_i_* represents the actual value, *y* represents the model’s predicted value, *N* is the sample size, and ${\bar{y}}_{i}$ is the average value of the actual values.

## 4. Conclusions

This study addresses the core issues of a single ML model architecture and insufficient interpretability in the prediction of bandgaps for LFDPs. Based on 2367 valid data from the open database of the Materials Project and related literature, after data preprocessing and feature engineering such as MinMaxScaler normalisation and Pearson correlation filtering, the data were divided into training set, validation set, and test set in a ratio of 7:1:2. Four deep learning models, namely MLP, deep ensemble learning, PINN, and Transformer, were used to predict the bandgaps of LFDP-Os. The performance was compared, and the importance of features was analysed using the SHAP method. The results show that the MLP model performs optimally in regression tasks involving medium-scale and structured features for bandgap prediction. Its test set R^2^ reaches 0.9311, while MAE, MSE, and RMSE are 0.1915 eV, 0.0975 eV^2^, and 0.3122 eV, respectively. It achieves a prediction error of ≤0.4 eV for 98% of the test samples, especially showing outstanding stability in low bandgap systems. On the other hand, the Transformer is more suitable for material performance prediction involving large-scale and sequential features. Additionally, the study found that the MLP model has limited generalisation ability for medium–high bandgap systems containing special elements such as Si and Mg. The SHAP analysis revealed that the five electronic structure descriptors, B_HOMO+, A_LUMO+, B_X+, A_X+, and A_IE+, are key regulators of the bandgap. They play a dominant role in shaping the bandgap from multiple dimensions and provide a physical basis for the targeted regulation of the bandgap. Based on this, in perovskite design, these descriptors can be targeted to achieve precise design and optimisation of the bandgap.

Regarding the generalisation shortcomings of the MLP model in systems containing special elements such as Si and Mg, future research will further expand experimental and theoretical data, and introduce strategies such as transfer learning and small sample learning to enhance the model’s universality; at the same time, by combining low-dimensional material data with quantum confinement-related features, the application scope of the model in low-dimensional LFDP systems will be expanded. Future work can also combine the k-space information in the energy band structure to explore the possibility of predicting direct/indirect bandgaps, and further improve the material performance evaluation system.

## Figures and Tables

**Table 1:** Descriptors for bandgap characteristics of LFDP-Os.

Feature	Unit	Description
A_OS, A’_OS	None	The oxidation states of the elements at positions A and A’
A_HOMO−, A_HOMO+	eV	The energy of the highest occupied molecular orbital (HOMO) of element A
A_IE−, A_IE+	kJ/mol	Ionisation energy of element A
A_LUMO−, A_LUMO+	eV	The lowest unoccupied molecular orbital (LUMO) energy of the A position element
A_X−, A_X+	None	Electronegativity of element A
A_Z_radii−, A_Z_radii+	a.u.	The Zunger pseudopotential radius of element A
A_e_affin−, A_e_affin+	kJ/mol	Electronic affinity of element at position A
B_OS, B’_OS	None	The oxidation states of the elements at positions B and B’
B_HOMO−, B_HOMO+	eV	The B element occupies the highest energy level of the molecular orbital (HOMO)
B_IE−, B_IE+	kJ/mol	Ionisation energy of the B element
B_LUMO−, B_LUMO+	eV	The lowest unoccupied molecular orbital (LUMO) energy of the B-type element
B_X−, B_X+	None	Electronegativity of the B element
B_Z_radii−, B_Z_radii+	a.u.	The radius of the Zunger pseudopotential for the B element
B_e_affin−, B_e_affin+	kJ/mol	The electron affinity of the B element
μ	None	Octahedral factor
μ$\overset{-}{A}$	None	A-position mismatch factor
μ$\overset{-}{B}$	None	B-position mismatch factor
τ	None	Tolerance factor

**Table 2:** Group with strong correlation between bandgap and features.

Feature A	Feature B	Correlation Coefficient
B_HOMO+	B_IE+	−0.8536
B_HOMO−	B_IE−	0.8050
B’_OS	B_IE−	0.8050
A_IE+	A_X+	0.8330
B’_OS	B_HOMO−	1.0000

**Table 3:** Comparison of performance results of the bandgap assessment regression model.

Algorithm	Evaluation Indicators			
	MAE	MSE	RMSE	R^2^
Deep ensemble learning	0.2287	0.1183	0.3440	0.9163
MLP	0.1915	0.0975	0.3122	0.9311
PINN	0.2370	0.1353	0.3678	0.9044
Transformer	0.3591	0.2577	0.5076	0.8178

**Table 4:** Bandgap prediction results of the MLP model on the test set.

LFDP-Os Order Number	Functional Group	PBE Bandgap (eV)	Prediction Bandgap (eV)	Prediction Error (eV)
1	AgCdReAuO_6_	0.0215	0.0344	−0.0129
2	AgTaO_3_	1.7301	1.5509	0.1792
3	AsBO_3_	0.5587	1.0036	−0.4449
4	AuBaMnInO_6_	0.0058	0.0223	0.0165
5	Ba_2_BiCeO_6_	0.0110	0.1021	−0.0911
6	Ba_2_BiCrO_6_	0.0072	0.0672	−0.0600
7	Ba_2_BiDyO_6_	1.7309	1.7762	−0.0453
8	Ba_2_CdTeO_6_	0.7433	0.9496	−0.2063
9	Ba_2_DySbO_6_	3.4682	3.5536	−0.0854
10	Ba_2_DyWO_6_	0.0414	0.0280	0.0134
11	Ba_2_ErBiO_6_	1.6093	1.5892	0.0201
12	Ba_2_ErSbO_6_	3.3728	3.4768	−0.1040
13	Ba_2_GaNbO_6_	3.0858	3.2184	−0.1326
14	Ba_2_GdTaO_6_	3.1809	3.3374	−0.1566
15	Ba_2_HfSiO_6_	4.0303	3.1283	0.9020
16	Ba_2_HfZrO_6_	3.2399	3.1877	0.0522
17	Ba_2_InSbO_6_	0.4948	0.0935	0.4013
18	Ba_2_MgTeO_6_	1.7447	2.5726	−0.8279
19	Ba_2_NbHoO_6_	2.8549	2.4990	0.3559
20	Ba_2_NdTaO_6_	3.3132	3.2104	0.1028

**Table 5:** Prediction results of MLP models in different bandgap range groups on the test set.

Bandgap Range (eV)	Sample Size	MAE (eV)	MSE (eV^2^)	RMSE (eV)	Error ≤ 0.4 eV Percentage
Low bandgap (0–1.0)	214	0.1915	0.0975	0.3122	91.12%
Medium–low bandgap (1.0–2.0)	97	0.1922	0.0979	0.3128	81.44%
Medium–high bandgap (2.0–3.5)	153	0.1932	0.0990	0.3146	86.27%
High bandgap (>3.5)	9	0.1942	0.0983	0.3135	66.67%

## Data Availability

The original contributions presented in this study are included in the article. Further inquiries can be directed to the corresponding author.
